# Subchondral bone microstructural damage by increased remodelling aggravates experimental osteoarthritis preceded by osteoporosis

**DOI:** 10.1186/ar3103

**Published:** 2010-08-02

**Authors:** Miriam Bellido, Laura Lugo, Jorge A Roman-Blas, Santos Castañeda, Jose R Caeiro, Sonia Dapia, Emilio Calvo, Raquel Largo, Gabriel Herrero-Beaumont

**Affiliations:** 1 Bone and Joint Research Unit, Service of Rheumatology. Fundación Jiménez Díaz, Universidad Autónoma. Avda. Reyes Católicos, 2. 28040 Madrid, Spain; 2 Department of Rheumatology. Hospital de la Princesa, Universidad Autónoma. Calle de Diego de León, 62. 28006 Madrid, Spain; 3 Trabeculae S.L. Parque Tecnológico de Galicia. 32900 San Cibrao das Viñas, Ourense, Spain; 4 Department of Orthopaedic Surgery. Fundación Jiménez Díaz, Universidad Autónoma. Avda. Reyes Católicos, 2. 28040 Madrid, Spain

## Abstract

**Introduction:**

Osteoporosis (OP) increases cartilage damage in a combined rabbit model of OP and osteoarthritis (OA). Accordingly, we assessed whether microstructure impairment at subchondral bone aggravates cartilage damage in this experimental model.

**Methods:**

OP was induced in 20 female rabbits, by ovariectomy and intramuscular injections of methylprednisolone hemisuccinate for four weeks. Ten healthy animals were used as controls. At week 7, OA was surgically induced in left knees of all rabbits. At 22 weeks, after sacrifice, microstructure parameters were assessed by micro-computed tomography, and osteoprotegerin (OPG), receptor activator of nuclear factor-κB ligand (RANKL), alkaline phosphatase (ALP) and metalloproteinase 9 (MMP9) protein expressions were evaluated by Western Blot at subchondral bone. In addition, cartilage damage was estimated using the histopathological Mankin score. Mann-Whitney and Spearman statistical tests were performed as appropriate, using SPSS software v 11.0. Significant difference was established at *P *< 0.05.

**Results:**

Subchondral bone area/tissue area, trabecular thickness and polar moment of inertia were diminished in OPOA knees compared with control or OA knees (*P *< 0.05). A decrease of plate thickness, ALP expression and OPG/RANKL ratio as well as an increased fractal dimension and MMP9 expression occurred at subchondral bone of OA, OP and OPOA knees vs. controls (*P *< 0.05). In addition, the severity of cartilage damage was increased in OPOA knees vs. controls (*P *< 0.05). Remarkably, good correlations were observed between structural and remodelling parameters at subchondral bone, and furthermore, between subchondral structural parameters and cartilage Mankin score.

**Conclusions:**

Microstructure impairment at subchondral bone associated with an increased remodelling aggravated cartilage damage in OA rabbits with previous OP. Our results suggest that an increased subchondral bone resorption may account for the exacerbation of cartilage damage when early OA and OP coexist simultaneously in same individuals.

## Introduction

Osteoarthritis (OA) is a multidimensional disease that affects all anatomical joint structures, particularly cartilage and subchondral bone [1,2]. In turn, osteoporosis (OP) is a skeletal disorder characterized by a compromised bone strength which substantially increases the risk of fracture [3]. Although OP and OA are two of the most prevalent skeletal disorders, both diseases are not frequently present in the same patient suggesting a mutually exclusive relationship. However, clinical studies often provide contradictory results which do not contribute to define the nature of the relationship between these medical conditions [4,5]. Furthermore, some confounding variables such as race, overweight and physical activity could explain this inverse relationship *per se *[6,7]. In addition, several studies may have been biased by design deficiencies, no radiological confirmation for OA diagnosis, bad patient positioning at X-ray assessment and/or the presence of osteophytes [8,9]. In this context, animal models experiencing both pathologies without interference from other factors have become valuable tools. Indeed, in combined models of low bone mass and OA by destabilization, ovariectomy alone or associated with glucocorticoid administration increased joint damage [10,11].

OA has been mostly considered a cartilage disorder. Nevertheless, the integrity of articular cartilage has been proposed to depend on mechanical properties of the underlying bone [12-14]. Several studies have recognized specific changes in the architecture and turnover of OA subcondral bone [15-17]. In fact, cartilage damage is frequently associated with thickening of the subchondral plate and osteophytosis during knee OA in humans [14-16,18] and animals [19]. But besides this hypertrophic OA, some authors contemplate another variant, the atrophic form, which is characterized by the lack of osteophytes and loss of subchondral bone volume in OA patients with hip and knee compromise [20-23]. Furthermore, the correlation between the serum levels of both C-propeptide and collagenase of type II collagen observed in hypertrophic OA was lost in atrophic OA, where exists a reduced type II collagen synthesis [24]. This could contribute to the absence of osteophyte formation, as well as to the increased subchondral bone turnover in rapidly progressive hip and knee OA [25,26]. Remarkably, the presence of subchondral bone attrition in knee OA, defined as flattening or depression of the osseous articular surface, is strongly associated with subchondral bone marrow lesions (BMLs) on MRI, In turn, BMLs reflect the presence of active remodeling processes due to chronic overload [23]. Cartilage loss occurred in the same knee subregions as subchondral bone attrition [27]. Likewise, subchondral bone in OA animal models with early stages of the disease have shown both decreased volume and stiffness [28,29], and increased remodelling [29]. The impact of these subchondral bone changes in OA is still debated, partially due to heterogeneity of the disease [30].

Different molecular alterations have been described to take place in the remodelling of OA subchondral bone [31]. In this regard, the receptor activator of nuclear factor-κB ligand (RANKL) and the osteoprotegerin (OPG), members of the RANKL/RANK/OPG pathway, are expressed differently whether the subchondral bone is affected or not by OA [32,33]. This pathway has been acknowledged as a key mechanism in the regulation of osteoclast formation or activation [34]. Although subchondral bone impairment seems to play a relevant role in OA onset and progression [31], the underlying mechanisms remain unknown. Therefore, an improved understanding of these mechanisms would increase our knowledge in the contribution of subchondral bone on cartilage damage and on its suitability as a therapeutic target in OA.

Thus, the present study aimed to assess whether microstructure impairment at subchondral bone may enhance cartilage damage when OA and OP are present simultaneously in rabbits. Furthermore, we characterize several bone microstructural parameters and metabolic modulators involved in subchondral remodelling as well as their relationships in these rabbits with OA aggravated by previous OP.

## Materials and methods

### **Animals**

Thirty skeletally mature female, eight-month-old (3.8 to 4.8 kg body weight), New Zealand rabbits were included in the study (Granja Universal, Pamplona, Spain). The animals became acclimatized after two weeks and were housed individually in stainless-steel cages and maintained on a 12-h light/12-h dark cycle at room temperature. The animals had free access to water and standard rabbit chow (Panlab, Barcelona, Spain). The guidelines for care and use of animals were followed throughout the study, in accordance with procedures approved by the Institutional Review Board.

### Experimental animal model

**Figure 1:**
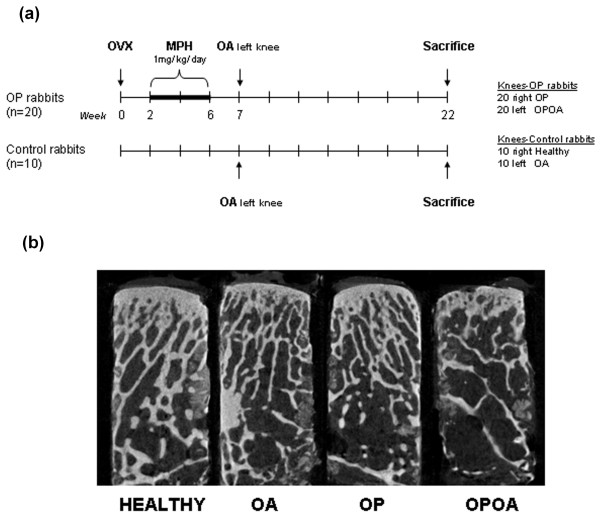
**Experimental design**. This study evaluated the effect of increased remodelling on subchondral bone microstructure and biomechanical-related genometrics in a combined rabbit model of osteoarthritis (OA) aggravated by previous osteoporosis (OP). **(a)**Experimental scheme. OVX: ovariectomy; MPH: methylprednisolone hemysuccinate. **(b)**Qualitative 2D models were reconstructed from images selected of a highly representative sample for each group (HEALTHY, OA, OP and OPOA), by using the CTAn software (Skyscan, Aartselaar, Belgium).

OP was induced in 20 rabbits, by ovariectomy and intramuscular injections of methylprednisolone hemisuccinate (1 mg/kg/day for four weeks; OP group). Ten age- and gender-matched additional animals were used as controls (healthy group). Surgical OA was induced in the left knees in all rabbits through medial meniscectomy and anterior cruciate ligament (ACL) section seven weeks after the beginning of the study. For ovariectomy and experimental OA, all rabbits were anesthetized and antibiotic prophylaxis was used according to protocol previously described [11]. Left knees were considered as OA (*n *= 10) or as OA and OP (OPOA; *n *= 20) and right knees were used as OP (*n *= 20) or healthy controls (*n *= 10), respectively. All animals were permitted free cage activity after surgery. Rabbits were euthanized by intracardiac administration of sodium pentobarbital (50 mg/kg; Pentotal, Abbott, Madrid, Spain) 15 weeks after knee surgery (Figure 1A). At sacrifice, samples of articular cartilage and subchondral bone of each knee were collected for further histological and Western Blot analysis or microstructural studies. Serum samples were taken for determining total alkaline phosphatase (ALP) and tartrate-resistant alkaline phosphastase (TRAP) activity assays at baseline and at 6, 12, 16 and 21 weeks after the study onset. All the experiments were approved by the local ethics committee.

### Subchondral bone microstructural study

Cylindrical biopsies of subchondral bone with the size of 9 mm in length by 4 mm in diameter were extracted from femoral condyles with a stainless steel trepan bur for explantation (Group Komet, Lemgo, Germany). The trepan bur was connected to a surgical motor (KaVo Dental GmbH, Biberach, Germany) that provided a speed of 1,500 rpm under continuous irrigation with saline soultion. Following this, the obtained biopsies were hydrated in saline solution and frozen at -20°C for subsequent analysis.

The microarchitecture of the samples was studied using micro-computed tomography (μCT; Skyscan 1172; Skyscan, Aartselaar, Belgium). The X-ray source was set at 100 kV and 100 μA, with a pixel size of 10.9 μm. Four hundred and fifty projections were acquired over an angular range of 180° (angular step of 0.40°). Image slices were reconstructed using the NRecon software (Skyscan, Aartselaar, Belgium) based on the Feldkamp algorithm, and applying corrections for beam hardening and ring artifacts. Registered datasets were segmented into binary images using global thresholding methods. The trabecular bone was extracted by drawing round contours with CTAn software (Skyscan, Aartselaar, Belgium) and the regions of interest selected were analyzed with the same application (see Figure 1B).

The quantitative structural two-dimensional (2D) variables determined for selected regions were: tissue area (T.Ar), bone area (B.Ar), bone area fraction (B.Ar/T.Ar%), trabecular thickness (Tb.Th), trabecular number (Tb.N) and trabecular separation (Tb.Sp). Furthermore, we assessed the thickness of the first layer of subchondral plate by measuring the distance between the subchondral bone edge and the point where the value of B.Ar/T.Ar drops to more than 98% of its value at the bone edge. This point would indicate the presence of subchondral trabecular bone. In addition, the fractal dimension (FD) and polar moment of inertia (Ip) were calculated for each sample. FD was measured as complexity index of the bone microarchitecture [35], and calculated using the Kolmogorov or *box counting *method by the Skyscan CTAn software. For 2D calculation of FD, the cross-sectional image was divided into an array of equal squares, and the number of squares containing part of the bone surface is counted. This was repeated over a range of square sizes such as 2 to 100 pixels. The number of squares containing the bone surface is plotted against square length in a log-log plot, and finally FD was obtained from the slope of the log-log regression. In turn, Ip was assessed as geometric index of bone strength to resist torsion [36]. Furthermore, the moment of inertia represents the rotational analogue of mass for linear motion. For a point mass (represented by an image pixel) the moment of inertia (I) is the mass (m) times the square of perpendicular distance (r) to the rotation axis, I = mr^2^.

### Western Blot analysis

For OPG, RANKL, ALP and MMP9 protein expression studies, 40 μg of total protein from tibial subchondral bone were loaded and then resolved on 10% acrylamide-SDS gels. After transfer to polyvinylidene difluoride (PVDF) membranes (Millipore, Molsheim, France), membranes were blocked in 5% skimmed milk in PBS-Tween 20 for 1 h at room temperature, and incubated overnight at 4°C with primary antibodies. These antibodies included goat anti-OPG (R&D Systems, Abingdon, UK) at 1/1000 dilution, rabbit anti-sRANKL (Prepotech, Neuilly-Sur-Seine, France) at 1/500, mouse anti-ALP (Abcam, Cambridge, UK) at 1/500, and mouse anti-MMP9 (Calbiochem/Merck Chemicals, Nottingham, UK) at 1/500. Later, antibody binding was detected by enhanced chemoluminiscence using peroxidase-labelled secondary antibodies and densitometric results were expressed in arbitrary units (AU) normalized to Glyceraldehyde 3-phosphate dehydrogenase (GAPDH) levels.

### Serum total ALP and TRAP activity assay

Serum ALP activity was determined by colorimetry, using p-nitrophenyl phosphate as substrate in a glycine buffer (glycine 50 mM; Cl_2_Mg 0.5 mM, pH 10.5). Serum samples were incubated at 37°C during 60 minutes, and then the hydrolysis reaction was stopped with NaOH 0.02N. Finally, absorbance was measured at 405 nm, since the serum ALP activity is directly proportional to the amount of substrate degraded. For the TRAP kinetic assay, the method was similar but the samples were incubated in a sodium acetate 50 mM and sodium tartrate 10 mM (pH 4.8) buffer and the reaction was stopped with NaOH 0.1N.

### Cartilage histology

After sacrifice, femur and tibia sections were fixed in buffered formalin for 24 h and then decalcified for six weeks in an ethylenediaminetetraacetic acid (EDTA) solution (2 mM EDTA, 0.5 mM tartrate sodium potassium, pH 1) for further histological evaluation.

The decalcified knee joints were cleaved in a sagital plane along the central portion of the articular surface of each medial femoral condyle corresponding to the weightbearing area, before embedding in paraffin wax. Cartilage sections (5 μm) were stained with hematoxylin and eosin to assess cellularity and structural abnormalities, and with Alcian blue to evaluate matrix abnormalities. The weight-bearing area of the femoral condyle was delimited and histopathologically assessed using the Mankin's grading system [37] by an experienced cartilage pathologist. The observer was blinded with respect to the rabbit group, laterality and macroscopic description. Samples were presented to the observer in random order. A partial score for each scale category (structure abnormalities, cellularity, matrix staining and tidemark integrity) was allocated, and the scores in each of these categories were combined for every section. The evaluation was performed at the weight bearing surface of the medial femoral condyle because it shows the earliest and most severe histological abnormalities [11,38].

### Statistical analysis

Results are expressed as mean ± standard error of the mean (SEM). Data from multiple groups were compared using Mann-Whitney non-parametric analyses as appropriate. Correlations were evaluated using the Spearman test. All statistical analyses were performed using commercially available software (SPSS v 11.0, SPSS Inc, Chicago, USA). Differences were considered significant when *P *< 0.05.

## Results

### Microstructure impairment at subchondral bone

**Figure 2:**
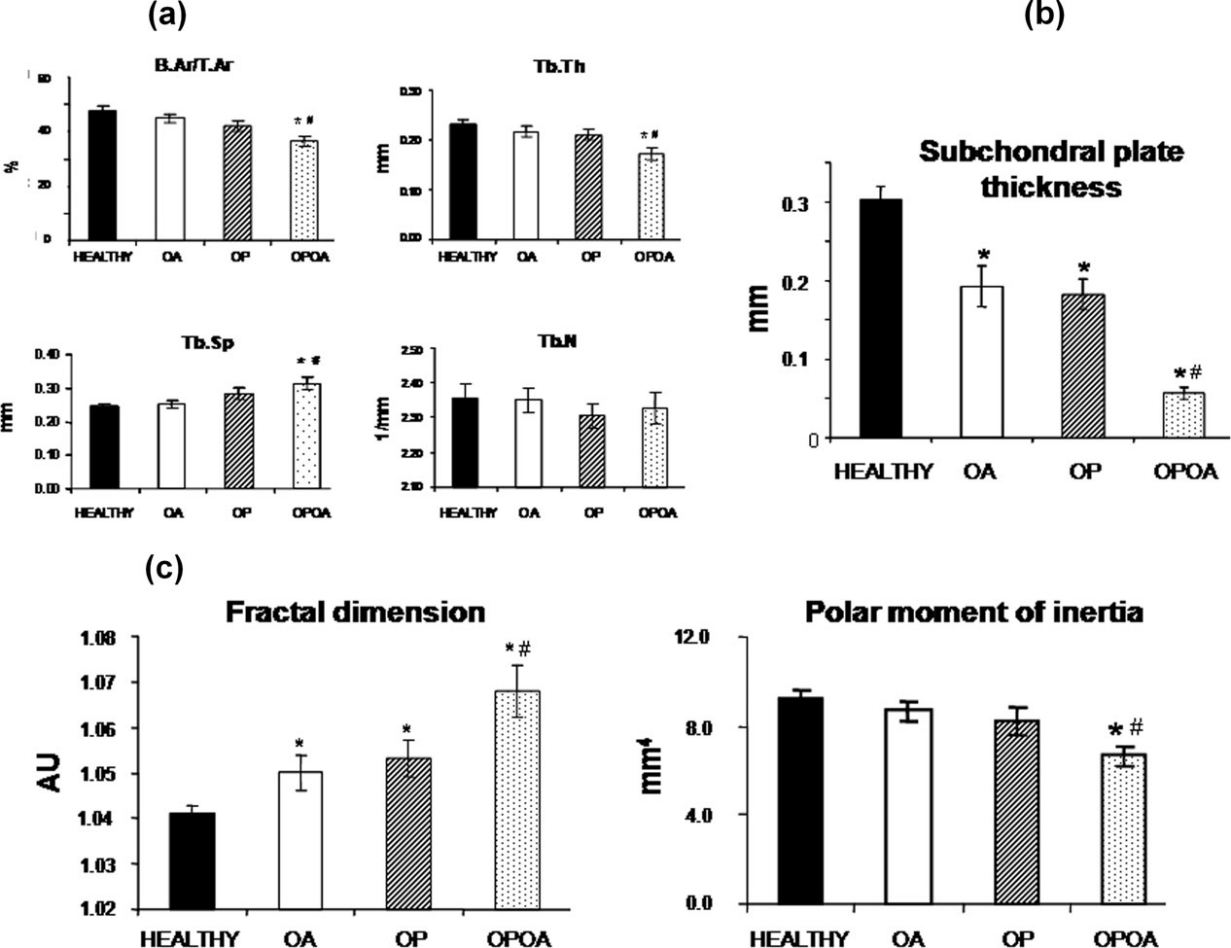
**Microstructure parameters at subchondral bone**. **(a)**Bone area fraction (B.Ar/T.Ar), trabecular thickness (Tb.Th), trabecular number (Tb.N) and trabecular separation (Tb.Sp) were assessed at subchondral bone. **(b)**Subchondral plate thickness. **(c)**Biomechanical-related structural parameters, fractal dimension (FD) and polar moment of inertia (Ip), at subchondral bone. HEALTHY (*n *= 7), OA (*n *= 7), OP (*n *= 8) and OPOA (*n *= 8) knees. OA, osteoarthritis; OP, osteoporosis. Values are expressed as mean ± SEM; *P < 0.05 vs. HEALTHY, #*P *< 0.05 vs. OA.

Changes in subchondral microstructure were studied 15 weeks after meniscectomy and ACL section of the knee to induce OA in a rabbit OP model (Figure 2A). Indeed, healthy knees showed a B.Ar/T.Ar higher than OPOA group (*P *< 0.05). Moreover, B.Ar/T.Ar in OPOA knees was decreased with respect to that in OA knees (*P *< 0.05). Similarly, Tb.Th in groups affected by OP or both conditions showed a downward trend with respect to healthy controls, however, only the combined OPOA group showed a significant decrease (*P *< 0.05). Conversely, Tb.Sp was increased in OP and OPOA knees with respect to healthy group (healthy: 0.24 ± 0.01 mm; OP: 0.28 ± 0.02 mm, and OPOA: 0.31 ± 0.02 mm), yet only OPOA significatively increased it (*P *< 0.05). Regarding Tb.N, no differences were found between the healthy and other groups (*P *> 0.05).

Notably, the plate thickness of subchondral bone was diminished in OA, OP or OPOA groups when compared with the healthy one (*P *< 0.05). Even more, the combined OPOA displayed a more decreased subchondral plate thickness than the OA group (*P *= 0.004), see Figure 2B. In addition, the microarchitecture index FD was increased in all groups *vs*. healthy (*P *< 0.05), and notably the OPOA group presented the highest difference against healthy or OA groups (Figure 2C). Furthermore, the biomechanical-related index Ip tended to be lower in both OA and OP groups than healthy knees, but they did not reach a statistical significance. However, Ip value of OPOA group was decreased when compared to healthy or OA groups (*P *< 0.05) (Figure 2C).

### ALP and MMP9 protein expression at subchondral bone

**Figure 3:**
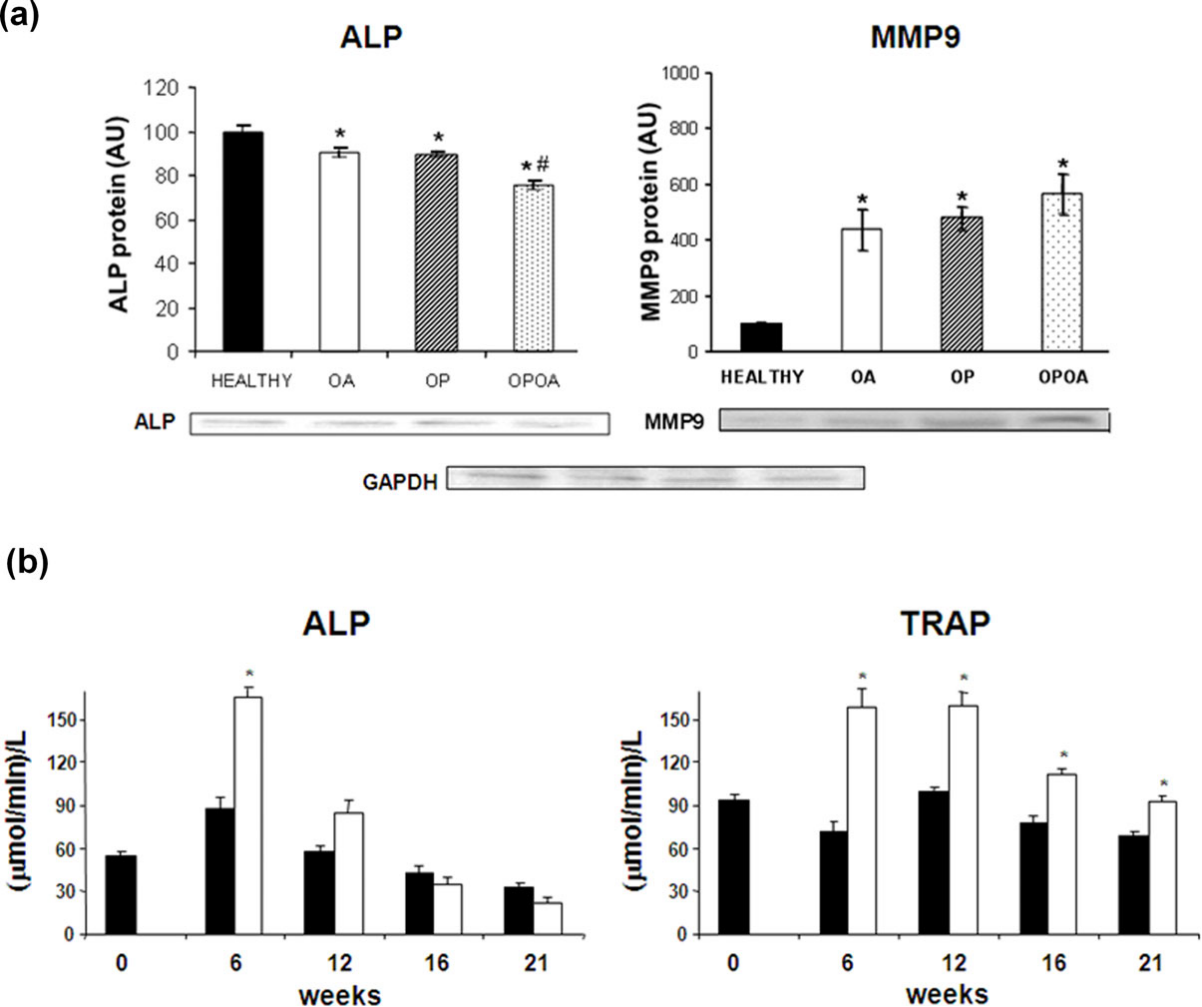
**Remodelling parameters at subchondral bone and systemic level**. **(a)**Alkaline hosphatise (ALP) and metalloproteinase 9 (MMP9) protein expressions at subchondral bone. At top: Densitometric analysis of ALP and MMP9 protein expressions at subchondral bone. At bottom: representative Western Blot images of ALP and MMP9 for 40 μg of total subchondral bone protein. The number of samples assayed for ALP expression were: HEALTHY *n *= 10; OA *n *= 8; OP *n *= 8 and OPOA *n *= 10, and for MMP9 were: *n *= 6 in each group. OA, osteoarthritis; OP, osteoporosis **(b)**Enzymatic activity for ALP and tartrato-resistant alkaline phosphatase (TRAP) in serum from OP animals (OP and OPOA groups; *n *= 8; white bars) and non-OP animals (healthy and OA groups; *n *= 7; black bars). OA, osteoarthritis; OP, osteoporosis. Bar represents the mean ± SEM. *P < 0.05 vs. HEALTHY, #*P *< 0.05 vs. OA.

Remodelling was estimated in each group by measuring the changes in protein expression of ALP and MMP9 at subchondral bone on week 22 (Figure 3A). Subchondral bone formation assessed by ALP protein expression was diminished in OA, OP and OPOA groups vs. healthy knees (healthy: 100 ± 3 AU; OA: 90 ± 1 AU; OP: 90 ± 2 AU; and, OPOA: 75 ± 2 AU; *P *< 0.05 in all cases). Furthermore, subchondral ALP expression in OPOA group also decreased with respect to that in OA group. In turn, subchondral bone degradation estimated by MMP9 protein expression was highly increased in OA, OP and OPOA groups with regard to healthy knees (*P *< 0.01 in all cases). However, although there was a tendency to increase, subchondral MMP9 expression in the combined group did not show a difference with respect to that in OA or OP groups.

### Increased serum ALP and TRAP enzymatic activities

In addition, serum enzymatic activities of ALP and TRAP were assayed in order to study the status of systemic bone turnover in our experimental animals. This analysis showed a high remodelling process in OP rabbits with or without OA (OP and OPOA groups), compared with those non-OP rabbits (healthy and OA groups). Indeed, an increase in both ALP and TRAP activity was found in OP and OPOA groups at week 6 (Figure 3B). Serum ALP activity was transiently increased until week 12 and restored to normal levels afterward in all groups. However, serum TRAP remained high throughout the study in OP rabbits with or without OA. It should be noted that systemic enzymatic activity measurements in OP or OPOA groups were the same since their knees come from same OP animals whether or not they underwent surgical OA. A similar situation occurred for healthy or OA groups that come from same non-exposed animals to OP stimulus.

### OPG/RANKL ratio at subchondral bone

**Figure 4:**
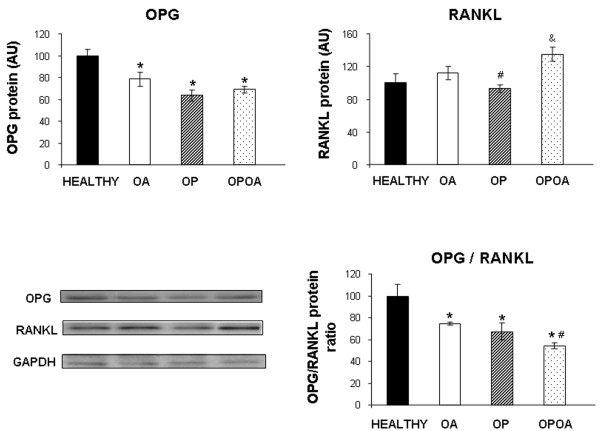
**Osteoprotegerin (OPG) and receptor activator of nuclear factor-κB (RANKL) protein expression at subchondral bone**. Densitometric analysis of OPG, RAKL and OPG/RANKL ratio, as well as, representative Western Blot images for OPG and RANKL are shown. To perform this blot, 40 μg of total protein from subchondral bone was used for HEALTHY, OA, OP and OPOA groups (*n *= 6, each group). OA: osteoarthritis; OP: osteoporosis. Data are displayed as arbitrary units ± SEM. *P < 0.05 vs. HEALTHY, #*P *< 0.05 vs. OA, &*P *< 0.05 vs. OP.

To determine the changes in RANKL/RANK/OPG pathway that occur in OA aggravated by previous OP, we have compared subchondral bone OPG and RANKL protein expression between groups by Western Blot (Figure 4). OPG synthesis was decreased in all experimental groups respect to healthy knees (healthy: 100 ± 6 AU; OA: 78 ± 6 AU; OP: 63 ± 5 AU, and OPOA: 69 ± 3 AU; *P *< 0.05 in all cases). RANKL expression was only increased in the OPOA group vs. healthy or OP groups (healthy: 100 ± 11 AU; OA:112 ± 8 AU; OP:93 ± 5 AU, and OPOA:135 ± 9 AU; *P *< 0.05). In addition, we have assessed the OPG/RANKL ratio finding a decrease in experimental groups when compared to healthy group. Furthermore, the combined group experienced a greater reduction in this ratio than OA group (*P *< 0.05).

### Cartilage histopathology

**Figure 5:**
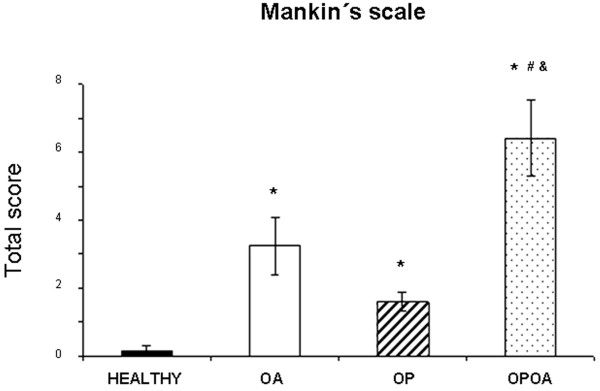
**Total Mankin score of histological cartilage damage**. The assessment was carried out at the weight bearing area of medial femoral condyle. HEALTHY (*n *= 7), OA (*n *= 7), OP (*n *= 8) and OPOA (*n *= 8) knees. OA: osteoarthritis; OP: osteoporosis. Results are expressed as mean ± SEM; *P < 0.05 vs. HEALTHY, #*P *< 0.05 vs. OA, &*P *< 0.05 vs. OP.

The impact of previous OP upon cartilage integrity in OA knees was estimated by using the histopathological Mankin grading score, see Figure 5. Thus, OPOA knees showed higher total Mankin scores than control knees (*P *< 0.05). Moreover, there were also differences when the scores obtained from OPOA knees were compared with OA knees. Overall Mankin scores in OA knees were higher than those in OP knees, but the differences were not significant. However, OP knees showed higher scores than normal healthy knees (*P *< 0.05), suggesting that OP could have an aggravating detrimental effect in the development of OA lesions.

### Correlations between structural and remodelling parameters at subchondral bone with cartilage damage

The associations between remodelling and structural parameters or OPG/RANKL ratio at subchondral bone were assessed in the different groups. Spearman correlations analysis (Table 1) showed that B.Ar/T.Ar% was directly associated with plate thickness and Ip at subchondral bone (*P *< 0.05), whereas, FD was inversely associated to the other structural parameters (*P *< 0.05). In the same way, at subchondral bone, the relationships were significant between ALP and the structural parameters B.Ar/T.Ar%, Ip or FD as well as between MMP9 and OPG/RANKL ratio. Moreover, there were significant correlations between subchondral bone structural parameters or ALP expression and cartilage damage.

**Table 1:** Associations between structural, remodelling and molecular parameters at subchondral bone, and cartilage damage score

	B.Ar/T.Ar%	Sb. Th	FD	Ip	ALP	MMP9	OPG/RANKL	MANKIN
**B.Ar/T.Ar%**	1.000							
**Sb. Th**	0.381*	1.000						
**FD**	-0.861**	-0.450*	1.000					
**Ip**	0.963**	0.347	-0.868**	1.000				
**ALP**	0.814**	0.453	-0.754**	0.718**	1.000			
**MMP9**	-0.115	-0.195	0.262	-0.094	-0.311	1.000		
**OPG/RANKL**	-0.280	-0.120	0.340	-0.247	-0.054	0.500*	1.000	
**MANKIN**	-0.627**	-0.626**	0.672**	-0.598**	-0.788**	0.460	0.341	1.000

* Correlation is significant at the 0.05 level (2-tailed).

** Correlation is significant at the 0.001 level (2-tailed).

The correlation coefficients represented in this table are obtained comparing the healthy, OA, OP and OPOA groups by Spearman bivariate analysis.

B.Ar/T.Ar%, bone area fraction; FD, fractal dimension; Ip, polar moment of inertia; MANKIN, cartilage histopathological Mankin grading scorel; MMP9, metalloproteinase 9; OA, osteoarthritis; OP, osteoporosis; OPG/RANKL ratio, osteoprotegerin/receptor activator of nuclear factor-κB ratio; Sb.Th, subchondral plate thickness.

## Discussion

We have studied the alterations in the microstructure of subchondral bone originated by an increased local bone resorption, as well as their relevant relationships with cartilage damage in the knees of OA rabbits with previous OP. Most of clinical studies suggest an inverse relationship between OP and OA. However, a negative OP effect upon articular cartilage integrity has been described in animal models [10,39-41]. Cartilage damage has been recognized not only by an impairment of structural and biomechanical properties, but also by identifying changes in matrix composition or collagen degradation in these OP models. In this sense, our group has reported that previous OP induces cartilage injury in a combined rabbit model of OP and OA [11].

In the current study, the knees presenting simultaneously OP and OA exhibited the thinnest subchondral plate, decreased B.Ar/TAr and Tb.Th, as well as increased Tb.Sp. In turn, the rabbits belonging to OP and OA groups showed a decrease in subchondral plate thickness and only a negative tendency in B.Ar/TAr and Tb.Th values. The decrease of subchondral plate thickness in OP, OA and particularly its pronounced decline in OPOA knees would indicate that our experimental model exhibits a much more profound effect in subchondral cortical bone than in subchondral trabecular bone. In agreement with our results, similar findings were observed in both murine and canine models of OA [28,42,43]. However, a thickening of subchondral plate was described in the guinea pig OA model by partial meniscectomy [19] and in the ACL transection-induced OA in rat model [44,45]. It is worth noting that subchondral microstructure changes in latter models corresponded to an advanced OA stage [19,44,45]. In addition, we have performed a microstructure analysis of the subchondral bone in a cylinder sample until 9 mm deep, thus studying not only the first cortical layer, but also the trabecular bone beneath. Consequently, our differences in respect to other studies could be derived from both the stage of OA development and the subchondral region of analysis.

Bone is an anisotropic material since its mechanical properties vary in different directions. In this context, an estimated mechanical stiffness of the trabecular bone can be obtained by an indirect method that considers the combination of bone volume fraction and architectural anisotropy. This method, the FD analysis, assesses the directional anisotropy of trabecular bone [46]. Other studies have also revealed that 2D fractal analysis relates to three-dimensional parameters such as porosity and connectivity [35]. In this way, FD in the combined model was increased, suggesting that subchondral bone that supports the articular cartilage in OPOA knees is more porous than that in other groups. Using fractal analysis by macroradiographs, it has been described a localized OP in large human OA joints [47,48]. On the other hand, bone architectural efficiency to resist torsion loads can be evaluated by the Ip, which is a relevant biomechanical bone factor. Thus, the higher the Ip, the stronger and more resistant the bone is to bearing torsion loads. By contrast, lower Ip drives to less bone stiffness [49]. In our study, this factor was lower in the OPOA group respect to those in other groups, hence suggesting a higher fragility of the subchondral bone of OPOA knees.

Bone remodelling is a finely regulated process resulting in the coordinated resorption and formation of skeletal tissue. The increase of systemic ALP and TRAP levels at week 6 in OP animals with and without OA (OP and OPOA groups) confirmed the presence of a high remodelling process during early OP stages, immediately after ovariectomy and glucocorticoid administration. However, on later days, the formation/resorption balance was oriented toward the resorption as TRAP activity continued high in OP rabbits with or without OA. Our results are in agreement with previous studies that have shown increased subchondral ALP levels at 38 days after ovariectomy in rats [50]. Moreover, in our study, OP, OA, and OPOA groups presented a lower ALP expression at subchondral bone which reflects the systemic turnover changes seen at week 21. In contrast, other authors reported the enzymatic ALP activity as invariable in OP or increased in OA [51]. These differences could be due to the late OA stage studied in human joints [51], while ALP assays in animal models have been performed at an early OA stage [29]. Furthermore, the negative glucocorticoid effects on bone formation in our experimental model could be reflected in the decrease of subchondral ALP synthesis in OPOA knees.

MMP9 expression was further analyzed as an index of the prominent enzymatic degradation of the extracellular matrix of subchondral bone and cartilage exerted by the MMPs. Furthermore, the presence of subchondral bone resorption pits composed by MMP-producing cells derived form bone marrow has been evidenced and they contribute to cartilage degradation [52]. Previous studies have described an increased MMP9 protein/gene expression not only in the subchondral bone but also in articular cartilage in both human and experimental OA studies [45,53,54]. Accordingly, we found raised subchondral MMP9 protein levels in OP, OA and OPOA groups demonstrating a clear increase in local resorption. Furthermore, subchondral MMP9 levels may reflect the increased serum TRAP activity seen in OP animals with or without OA (OP and OPOA groups) at week 21. In addition, to our knowledge, this study shows for the first time MMP9 up-regulation in an OP experimental model, which supports the role of this enzyme in bone resorption during OP. Taken together, all these findings indicate that OP increases OA subchondral remodelling through stimulation of bone resorption, and suggest that this detrimental effect may be particular relevant at early OA.

Remarkably, the changes at subchondral microstructure and remodelling were supported by the results obtained at the molecular level in this study. In fact, the subchondral OPG/RANKL ratio was decreased in the OA, OP and OPOA groups when compared to controls, suggesting a clear increase in subchondral bone resorption in all these conditions. Moreover, OPOA showed a smaller OPG/RANKL ratio than OP or OA alone, therefore, indicating a greater subchondral bone resorption in the combined group. These results are in line with previous studies in OP and OA. RANKL is the principal regulator of bone resorption by promoting OC differentiation, activity, and survival. OPG is a decoy receptor that binds to RANKL inhibiting OC formation and survival [55]. A decrease in the OPG/RANKL ratio is involved in the development of post-menopausal or glucorticoid-induced OP, bone erosions of rheumatoid arthritis, and bone disease associated with malignant and non-malignant conditions [56]. A reduced OPG/RANKL ratio has been also found in subchondral OBs [57] and chondrocytes of OA patients [58,59]. Moreover, celecoxib increased the OPG/RANKL ratio in human OA cartilage [59]. Interestingly, a decrease in this ratio in serum of OA patients has been related to cartilage damage [60]. Thus, the OPG/RANKL ratio plays a significant role in OP and likely in OA.

The significant correlations between bone structural, remodelling and molecular parameters in our animal model indicate that alterations in systemic and local remodelling lead to changes at the structural and biomechanical level at subchondral bone in OP, OA and particularly in OPOA group. All these changes contribute to impairment in subchondral bone quality, and make this organ not able to receive and properly distribute loads from and/or to the articular cartilage. Moreover, the significant correlation between subchondral microstructure impairment and cartilage damage denotes that changes at subchondral bone aggravate cartilage damage. Although discrepancies have been raised about subchondral changes originated by the simultaneous presence of OP and OA in same individuals, our findings support a synergism of both diseases, particularly when early OA stage is assessed. In addition, it is possible that in post-traumatic OA, trabecular microarchitecture changes are different than in primary OA. In fact, a potential osteopenia by disuse due to decreased load bearing has been proposed during the early post-operative period in the canine ACL-deficient knee [61].

## Conclusions

Thus, although obvious differences exist between idiopathic OA in humans and our rabbit mechanical model of the disease, the results of this work strengthen the role of the subchondral bone as a key player in the puzzle of OA development [12,31,42-45]. Also, they support our previous research [11] by demonstrating that microstructure impairment at subchondral bone associated with an increased remodelling increases cartilage damage in OA rabbits with previous OP. Precisely, in light of these studies, our current findings suggest that an increased subchondral bone resorption may account for the aggravation of cartilage damage when early OA and OP coexist simultaneously in the same individuals.

## Abbreviations

2D: two-dimensional; ACL: anterior cruciate ligament; ALP: alkaline hosphatise; AU: arbitrary units; B.Ar: bone area; B.Ar/T.Ar%: bone area fraction; EDTA: ethylenediaminetetraacetic acid; FD: fractal dimension; GAPDH: Glyceraldehyde 3-phosphate dehydrogenase; I: moment of inertia; Ip: polar moment of inertia; m: mass; MMP9: metalloproteinase-9; OPG: osteoprotegerin; PBS: phosphate buffer saline; PVDF: polyvinylidene difluoride; r: perpendicular distance; RANK: receptor activator of nuclear factor-κB; RANKL: receptor activator of nuclear factor-κB ligand; SDS: sodium dodecyl sulphate; SEM: standard error of the mean; SPSS: statistical package for the social sciences; Tb.N: trabecular number; Tb.Sp: trabecular separation; Tb.Th: trabecular thickness; TRAP: tartrate-resistant alkaline phosphastase; μCT: micro-computed tomography

## Competing interests

The authors declare that they have no competing interests.

## Authors' contributions

RL and GH-B designed and conceived of the study. MB, LL, JRC and DP acquired the data. MB, LL, JAR-B, SC, RL and GH-B analysed and interpreted the data. MB, LL, JA-RB, SC and GH-B drafted the manuscript. All authors revised the manuscript critically for important intellectual content, and all authors approved the final version to be published. GH-B had full access to all of the data in the study and takes responsibility for the integrity of the data and the accuracy of the data analysis.
